# Calendar ageing modelling using machine learning: an experimental investigation on lithium ion battery chemistries

**DOI:** 10.12688/openreseurope.14745.1

**Published:** 2022-08-12

**Authors:** Burak Celen, Melik Bugra Ozcelik, Furkan Metin Turgut, Cisel Aras, Thyagesh Sivaraman, Yash Kotak, Christian Geisbauer, Hans-Georg Schweiger

**Affiliations:** 1 Bogazici University, Istanbul, Turkey; 2 AVL Research and Engineering Turkey, Istanbul, Turkey; 3 AVL List GMBH, Graz, 8020, Austria; 4 Technische Hochschule Ingolstadt, Ingolstadt, 85049, Germany

**Keywords:** lithium-ion batteries, calendar ageing, artificial neural network, machine learning, XGBoost

## Abstract

**Background: **The phenomenon of calendar ageing continues to have an impact on battery systems worldwide by causing them to have undesirable operation life and performance. Predicting the degradation in the capacity can identify whether this phenomenon is occurring for a cell and pave the way for placing mechanisms that can circumvent this behaviour.

**Methods:** In this study, the machine learning algorithms, Extreme Gradient Boosting (XGBoost) and artificial neural network (ANN) have been used to predict the calendar ageing data belonging to six types of cell chemistries namely, Lithium Cobalt Oxide, Lithium Iron Phosphate, Lithium Manganese Oxide, Lithium Titanium Oxide, Nickle Cobalt Aluminum Oxide and Nickle Manganese Cobalt Oxide.

**Results:** Prediction results with overall Mean Absolute Percentage Error of 0.0126 have been obtained for XGBoost algorithm. Among these results, Nickle Cobalt Aluminum Oxide and Nickle Manganese Cobalt Oxide type cell chemistries stand out with their mean absolute percentage errors of 0.0035 and 0.0057 respectively. Also, algorithm fitting performance is relatively better for these chemistries at 100% state of charge and 60°C temperature compared to ANN results. ANN algorithm predicts with mean absolute error of approximately 0.0472 overall and 0.0238 and 0.03825 for Nickle Cobalt Aluminum Oxide and Nickle Manganese Cobalt Oxide. The fitting performance of ANN for Nickle Manganese Cobalt Oxide at 100% state of charge and 60°C temperature is especially poor compared to XGBoost.

**Conclusions:** For an electric vehicle battery calendar ageing prediction application, XGBoost can establish itself as the primary choice more easily compared to ANN. The reason is XGBoost’s error rates and fitting performance are more usable for such application especially for Nickel Cobalt Aluminum Oxide and Nickel Manganese Cobalt Oxide chemistries, which are amongst the most demanded cell chemistries for electric vehicle battery packs.

## Introduction

Lithium-ion (Li-ion) batteries are becoming the most promising key component for the vision of a sustainable future with the rise of e-mobility. It has earned this well-deserved reputation due to its high power and energy density and long cycle life ^1^. Although their proven success, as any other system does, Li-ion batteries also suffer from degradations over time because of different ageing phenomenons such as cyclic and calendar ageing. While both ageing mechanisms have their similarities in terms of the resulting electrochemical reaction, which is, simply put, the growth of solid electrolyte interface on the anode particle of battery ^2^, calendar ageing occurs during storage ^3^ while the cyclic ageing occurs during the deintercalation and intercalation of lithium due to the operation (charging-discharging) of the battery ^4^. In the end, both ageing mechanisms will result in decreased capacity ^3^ and increased resistance ^5^. In this paper, the focus will be on the capacity degradation due to the calendar ageing.

As mentioned, the calendar ageing of a battery occurs during storage, but the magnitude of the phenomenon is determined by three main factors: the rest state of charge (SOC), the rest temperature and the duration of the rest time of the Li-ion battery ^6^.

The recent studies conducted by Geisbauer *et al.*, proved that despite Li-ion technology offering a wide range of chemistries, such as Nickle Manganese Cobalt Oxide (NMC), Nickle Cobalt Aluminum Oxide (NCA), Lithium Titanium Oxide (LTO), Lithium Cobalt Oxide (LCO) or Lithium Iron Phosphate (LFP), none of them were able to avoid the calendar ageing ^7^. The study also clearly demonstrated high temperatures lead to even higher degradations ^7^. These factors are important because, e.g. if the application is an electric vehicle, the vehicle will spend most of its lifetime parked and this period of time may include high rest temperatures.

In order to implement functionalities that prolong the lifetime of the battery, the ageing of the Li-ion cells should be accurately estimated. There are physical, empirical and semi-empirical modeling solutions that can describe the behaviour of cyclic and calendar ageing. However, the physical models require a great deal of physical-chemical knowledge, really complex modeling and computational effort ^6^. In the meantime, less complex semi-empirical models require lesser physical-chemical knowledge but are not as accurate compared to the physical model ^6, 8^. A method that can minimize the computational effort, eliminates the need for physical-chemical knowledge for different cell chemistries but is still as accurate as an electrochemical (physical) model was lacking. However, a solution to this problem has been proposed by many authors in the literature ^5, 6^. The overall solution is to employ advanced machine learning algorithms that can accurately estimate calendar ageing. Therefore, this paper considers such algorithms as the main methodology of this research. It also covers the missing aspect of market and literature that is the comparison of machine learning algorithms accuracy for a wide spectrum of chemistries such as NMC, NCA, LTO, LMO, LCO and LFP. The reasons to study Li-ion chemistries in this paper are, (a) due to the significant portion of the current energy storage system applications are based on Li-ion technology, and (b) the study aligns with the previous research of 7. Thus, this study can take full advantage of a calendar ageing dataset of a wide range of Li-ion chemistries. This will enable the validation of algorithms on a much wider sense.

Calendar ageing with the cyclic ageing comprises the complete ageing phenomenon for Li-ion cells, and validation of whether the estimation of the calendar ageing is accurate is highly crucial since the phenomenon does not only have an impact on state of health estimation but also on the SOC estimation as well. The inaccurate estimations of the ageing of the battery will result in inaccurate SOC estimations (e.g limit breaches at full capacity during the charge of the battery due to faulty SOC estimation can occur. SOC estimation algorithm can estimate the accumulated charge more than the actual accumulated charge if the actual usable capacity of the cell is lower due to the ageing. This scenario can easily be realized with an ageing estimation model with low accuracy that underestimates the capacity decrease of the battery). The battery system’s current limitation estimations will be affected by these wrong SOC estimations due to the remaining charge being an important input in defining the current limits in both charge and discharge directions ^9^.

Consequently, this chain of wrong estimations along with the mentioned non-accurate current limitations will speed up the ageing effects of the batteries ^10^. The resulting impractical system may easily diminish battery operation lifetime and increase the safety risks ^11^. Therefore, the variety and the size of the validation data and the variety and comparison of the employed coupled modelling activities play an important role in this paper and help to prove that the chosen methods would yield highly accurate results. Another benefit of this work is that it can shed light on future system implementations that don’t exclude the phenomenon of calendar ageing. Usable accuracy results drawn from this work can realize systems that take into account the other half of the medallion in terms of ageing, without the time-consuming parameterization or characterization test activities (this half of the medallion being the calendar and the other one being the cyclic ageing which is being implemented in various systems).

The coupled modelling activities that will be demonstrated and will be used for the aim of predicting the calendar ageing via capacity degradation of the cells by using some of the early test data are Extreme Gradient Boosting (XGBoost) and Artificial Neural Network (ANN). XGBoost is a decision tree based machine learning algorithm, while the ANN is an artificial adaptive system that uses its base elements, called neurons and connections, to transform its global inputs into a predicted output ^12^. The motivation behind the selection of these algorithms is that both methods are known for their ability to yield reliable results ^6, 13^ as well as, by employing these algorithms, there would be a means of comparing the prediction performances of machine learning algorithms on different chemistries.

## Methods

### Ageing setup, data acquisition and processing

The complete ageing procedure of the different cell chemistries was performed at Technische Hochschule Ingolstadt (THI). Mainly, the cells were calendar aged in temperature chambers (Vötsch VT 4011 and Vötsch VT 4021) at raised temperatures of 50°C, 60°C and 70°C and high, medium and low voltages. The detailed storage conditions are described in previous research ^7^. Before any fast ageing procedure was employed, the cells were validated for their functionality with a capacity check and pulse profile to validate the internal resistance. The cylindrical cells were mounted onto self-constructed wooden shelves to maximize the number of cells per climate chamber. Check-ups, during which the cells were taken out, measured for capacity and internal resistance after a fixed relaxation time, were established in regular intervals for the different storage conditions. Before returning the cells into the climate chambers, they were recharged to their dedicated storage SOC as described in the 7.

The instrumentation equipment (Neware BTS 4008-5V6A cell tester) had a measurement accuracy of ±0.05% ^7^. This means that at this level of accuracy, the instrument can induce a capacity measurement error of 10,925 A.s (ampere-second) and subsequently a small relative error of 0.121388889. Because of this reason, in this work, the prediction results from the machine learning algorithms are only trying to fit to the measurement data which may incorporate these small accuracy errors. The performances of the algorithms are also validated according to this measurement data with significantly small accuracy errors.

For a more detailed description of the data acquisition, please refer to 7. After the ageing was finished, the evaluated data ^14^ was prepared for the modelling to be performed at AVL, where also post-processing methods were applied to make the datasets more usable for the machine learning algorithms.

### Extreme Gradient Boosting (XGBoost)

**Figure 1.**
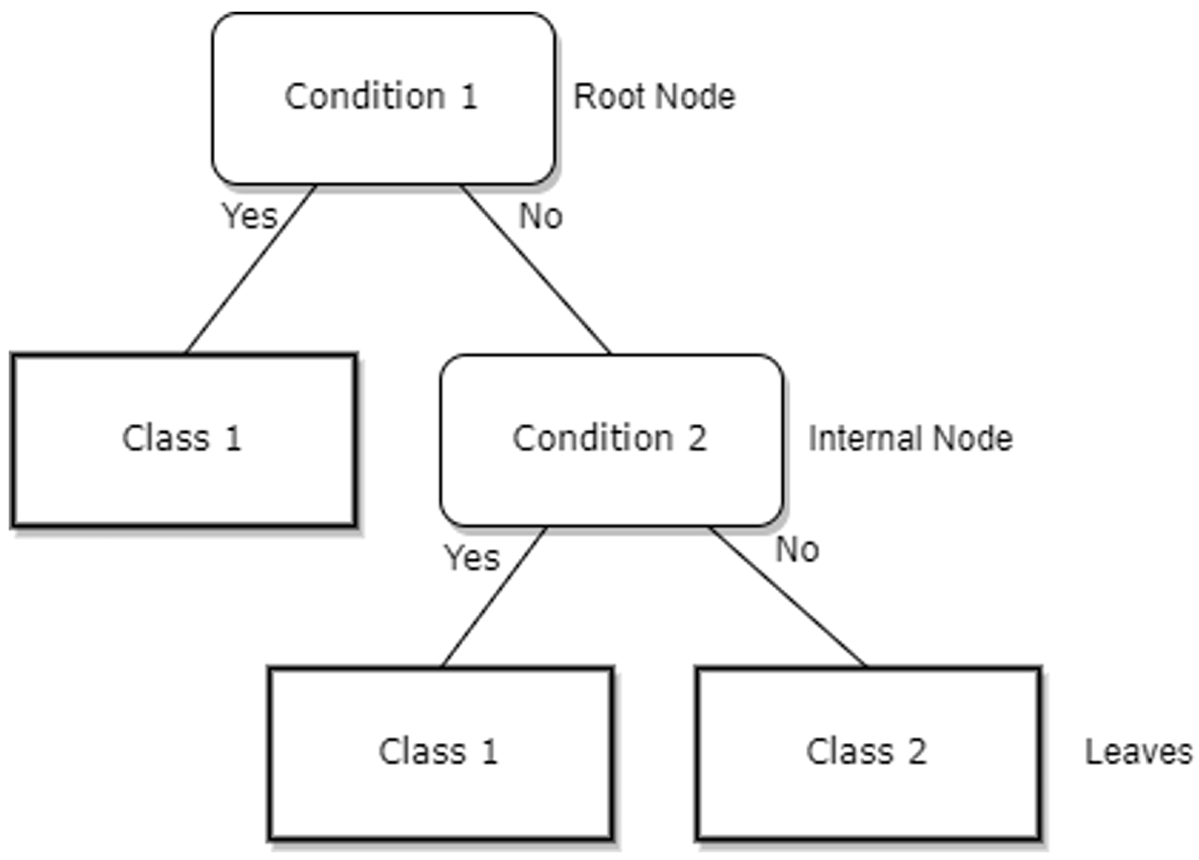
A simple decision tree.

Extreme Gradient Boosting (XGBoost) is a decision tree based, state-of-the-art supervised machine learning algorithm that is widely used in regression or classification problems. It proved its success by being the main solution technique of many machine learning competitions and problems in the industry ^15^. A decision tree consists of nodes, branches, and leaves. The algorithm starts with the root node and splits the data by branches according to the condition in it. Thereafter, it checks for the condition in the next node or makes the final decision in the leaves ^16^. Figure 1 demonstrates the example of the simple decision tree.

Gradient boosting algorithms are also based on decision trees. By gradient boosting methods, some weak learners - e.g. decision trees - can be transformed into strong learners. For regression tasks, a loss function is calculated for the results in the leaves, and the tree is reconstructed by calculating residuals ^17^. Therefore, the results are improved in every step of learning.

One of the main superiorities of XGBoost is its capability of being able to automatically handle null values in the dataset. In conventional machine learning tasks, data scientists must clear the null data before starting to build their learning algorithm. However, XGBoost can work on a dataset with null values and reduce the effort for data pre-processing to a great extent ^15^.

Also, XGBoost uses a greedy algorithm, weighted quantile sketch for finding the best learning score. It splits the data into instances like the quantile sketch algorithm. The improvement of XGBoost is that every portion of data is weighted ^15^.

Furthermore, XGBoost uses parallel computing with storing data in “column blocks”. These blocks are stored in the cache owing to its “cache-aware” system. The efficiency of this method depends on the size of blocks ^15^.

**Figure 2.**
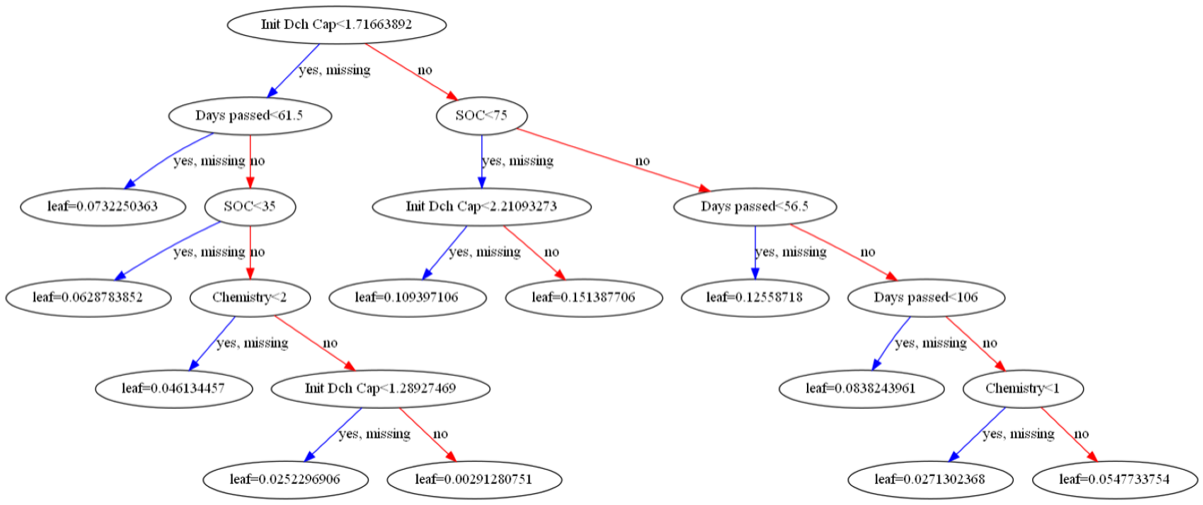
Calendar ageing XGBoost implementation decision tree.

For the calendar ageing problem, an XGBoost regressor is implemented to predict the capacity values. Figure 2 demonstrates the decision tree generated by the XGBoost algorithm.

It has been demonstrated in earlier studies that it is possible to deploy XGBoost models to embedded processors ^18^. This ability ensures the usage of this method for practical scenarios as well. Embedded control systems can take advantage of this ability to actualize the XGBoost implementations for a wider range of real-life problems.

### Artificial neural networks

In this work, artificial neural network (ANN) architecture is another method to model the calendar ageing in the battery cells. In ANNs, the modelling is done via adaptation of the individual weights based on the partial derivative of the error between the simulated output and desired output. At each cycle, the weights are adjusted to minimize the mentioned error.

**Figure 3.**
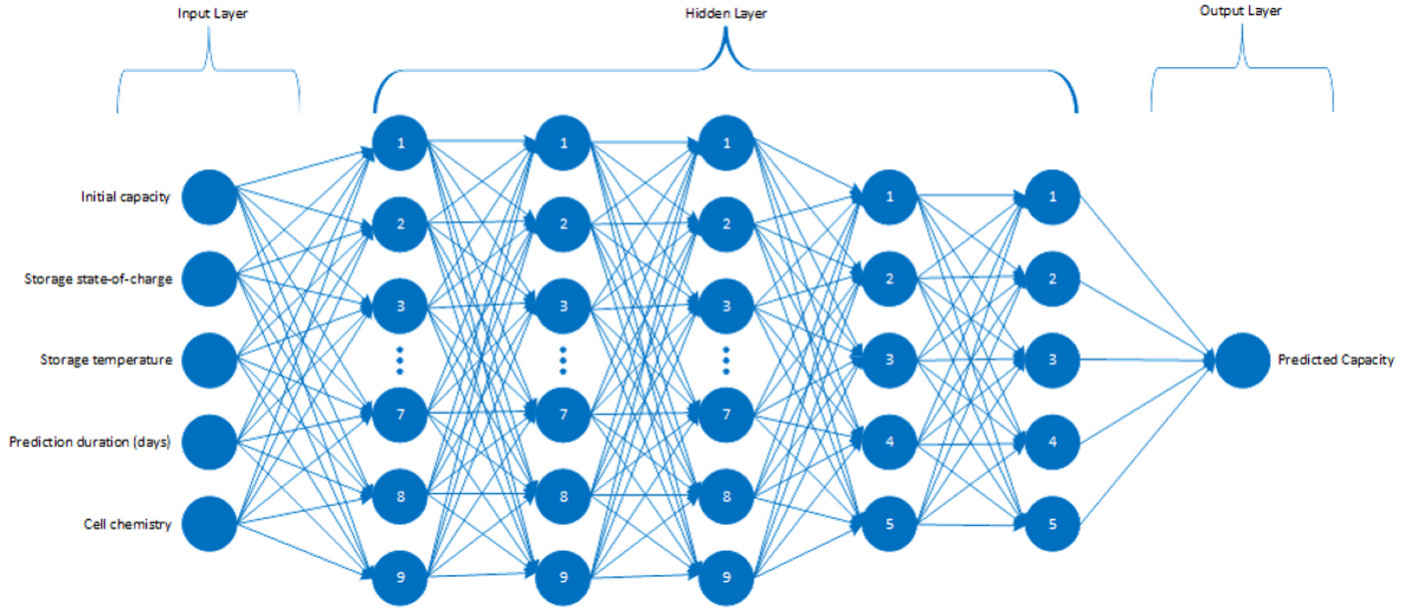
Implemented artificial neural network (ANN) structure.

In the end, the goal of this architecture is to diminish the error levels by estimating the function that will result with the desired output by any given input ^19^. In this study, the model is something rather conventional that maps a set of inputs to an output. The model’s architecture has five inputs that are listed as important features, five hidden layers with the sequence of 9,9,9,5,5 neurons and lastly, the output layer is predicting the capacity of the given cell considering the calendar ageing as a function of storage conditions like SOC and temperature, exposure duration in days to those conditions, specification of the Li-ion chemistry and brand-new capacity information of the cell. The described structure can be observed in Figure 3.

For the model, the results of each neuron can be mathematically described with the summation of the respected weights and inputs at the end activated with a special function known as Rectified Linear Unit (ReLU). This gives an output of 0 while the given input is smaller than zero (f(x) = 0 if x < 0) and output of itself while the input is either equal to zero or higher (f(x) = x if x => 0).


$$\;o_{j}=f(\sum_{n=1}^{N}w_{i,j}x_{i})\;(1)$$

In Equation 1, *N* is the number of inputs, *w* is the weights in between the neurons, *x* is the inputs forwarded to the next neuron. Here, *i* represents the enumeration of inputs, *j* represents the next layer’s enumeration of neurons. Finally, the output of the respected neuron is denoted as *o*.


$$\;E=\sum_{k=1}^{Q}o_{k}-y_{k}\;(2)$$

Updating the weights used in the equation or in other terms, estimating the function, is done via a technique called gradient descent. During the training process, the idea is to find the error equation described in Equation 2. Here, *Q* is the number of neurons, *o* is the activated neuron output, *y* is the actual output. The error is a function of weights, taking the partial derivative of the error equation with respect to weights and finally multiplying with an entity known as the learning rate *α* results in the update of weights as described in Equation 3.


$$\;w_{i,j}=-\alpha \frac{\partial E}{\partial w_{i,j}}\;(3)$$

During the training and verification steps, various configurations of hidden layers, neurons, learning rates and learning strategies are employed. Among them, normalizing the inputs around 0 to 1, setting hidden layers to 5 with 9,9,9,5,5 neurons and setting the learning rate ( *α*) to be constant 0.015 yielded the desirable outputs in the end. Later on, when needed, these results and studies can be used as a baseline to generate a system for embedded boards on battery management systems ^20^.

### Evaluation of results

In order to evaluate the performances of the algorithms, an evaluation metric has to be chosen. In this context, mean absolute error (MAE) along with its percentile variation mean absolute percentage error (MAPE) have been decided as the key performance indicators to benchmark two algorithms. MAE and MAPE are metrics that usually find applications to themselves in the machine learning field ^21^. Both MAE and MAPE determine the average magnitude of errors between predicted and measured values ^21^. The only difference is MAE is not unitfree which makes comparisons somewhat difficult, while MAPE doesn’t have this problem since it calculates based on the percentage error ^21^. This makes MAPE a better choice in the machine learning field to explain the relative errors ^21^. For both metrics, the same rule applies; the smaller the MAE/MAPE value, the higher the accuracy of prediction. The only disadvantage is, even though determining the magnitude of error gives an important insight, MAE or MAPE cannot provide the direction of error ^21^. The basic formulas for MAE and MAPE can be seen in equations Equation 4 and Equation 5.


$$MAE=\frac{1}{n}\sum_{1}^{n}\left|D_{pre}-D_{act}\right|\;(4)$$

$$MAPE=\frac{100\%}{n}\sum_{i=1}^{n}\left|\frac{D_{pre}-D_{act}}{D_{act}}\right|\;(5)$$

Here, *D _pre_* represents the predicted value and *D _act_* is the measured value. Meanwhile, *n* represents the number of result points.

## Results and discussion

**Figure 4.**
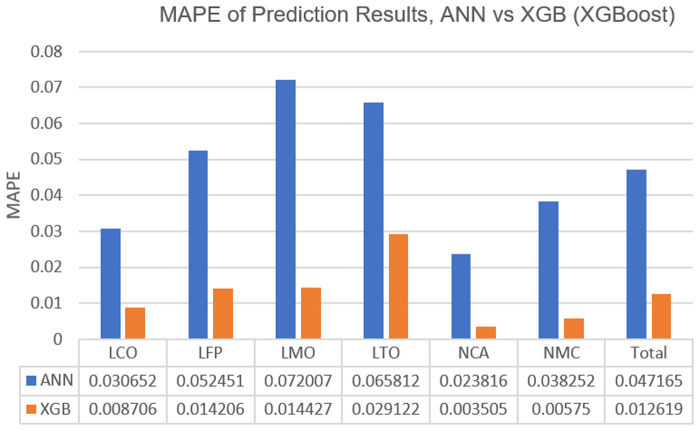
Bar graph of mean absolute percentage errors (MAPE) of Lithium Cobalt Oxide (LCO), Lithium Iron Phosphate (LFP), Lithium Manganese Oxide (LMO), Lithium Titanium Oxide (LTO), Nickle Cobalt Aluminum Oxide (NCA) and Nickle Manganese Cobalt Oxide (NMC) cell chemistries resulting from the artificial neural network (ANN) and Extreme Gradient Boosting (XGBoost) algorithms.

As mentioned in the introduction and methods, the algorithms have not been preferred just for achieving satisfactory results, but also because of the prospect of benchmarking and performance comparisons. For this reason, the results of both algorithms (XGBoost and ANN) were obtained for each cell chemistry in terms of MAPE and plotted as a bar graph in Figure 4 for clear inspection. Also, this graph has been supported with the corresponding ampere-hour (Ah) values to the MAPE values with Table 1. The first noticeable point from Figure 4 and Table 1 is that ANN is significantly underperforming for the prediction of capacity degradations due to the calendar ageing compared to the performance of XGBoost. Another point from this graph is the prediction performance of algorithms for cobalt included chemistries such as NMC, NCA and LCO, seems significantly better compared to their performances on the cobalt-free chemistries.

**Table 1.** Mean absolute error (MAE) values of Lithium Cobalt Oxide (LCO), Lithium Iron Phosphate (LFP), Lithium Manganese Oxide (LMO), Lithium Titanium Oxide (LTO), Nickle Cobalt Aluminum Oxide (NCA) and Nickle Manganese Cobalt Oxide (NMC) cell chemistries resulting from the artificial neural network (ANN) and Extreme Gradient Boosting (XGBoost) algorithms (in terms of ampere-hour).

Algorithms	LCO	LFP	LMO	LTO	NCA	NMC	Total
ANN	0.067567	0.076977	0.139979	0.076037	0.059225	0.092450	0.085372
XGB	0.018649	0.020621	0.028154	0.03472	0.008248	0.013664	0.020676

The superior performance of XGBoost on the chemistries that are dominating the automotive industry like NCA, NMC and LFP is another result that is highly convenient. NCA, NMC and LFP are comprising the most significant portion of the electric vehicle battery development projects ^22^, and this prediction performance brings forth the XGBoost’s feasibility for the automotive batteries’ calendar ageing prediction applications, combined with these algorithms’ prospects of deployment on embedded processors as mentioned in the methods.

**Figure 5.**
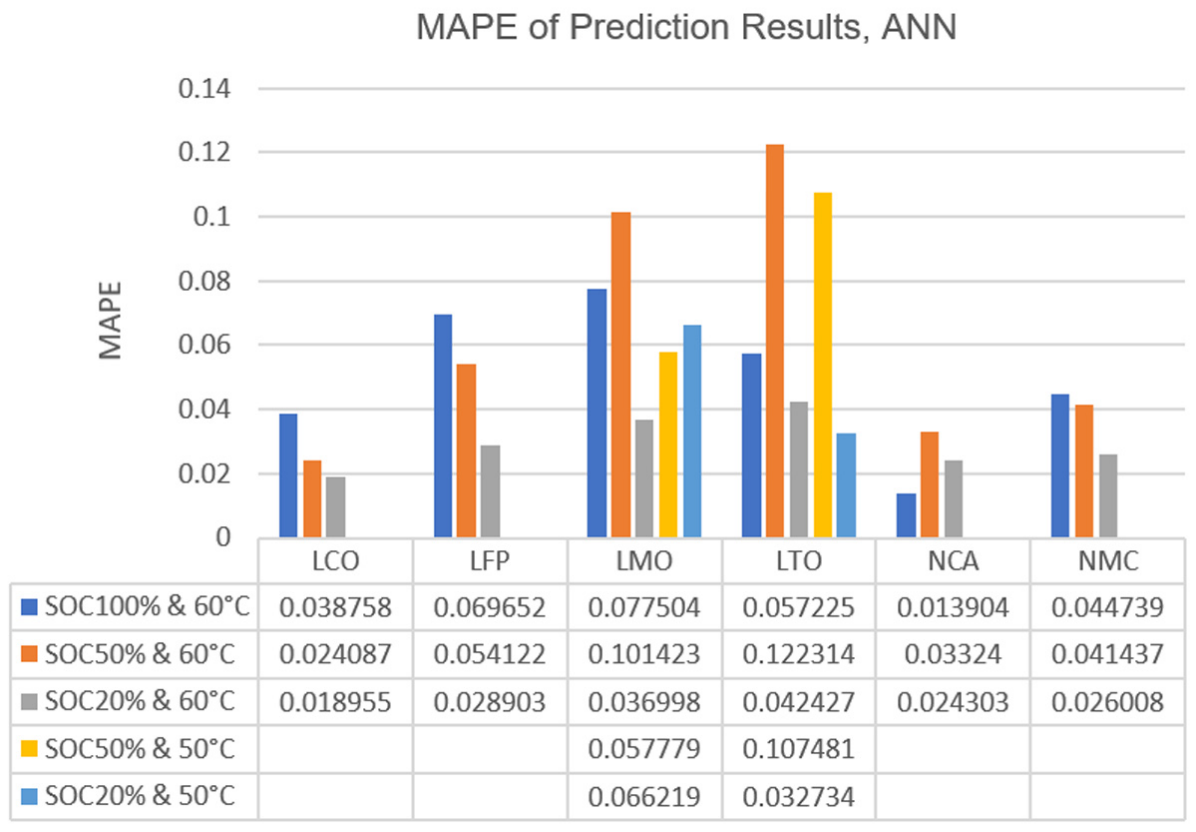
Bar graph of artificial neural network (ANN) mean absolute percentage errors (MAPE) of Lithium Cobalt Oxide (LCO), Lithium Iron Phosphate (LFP), Lithium Manganese Oxide (LMO), Lithium Titanium Oxide (LTO), Nickle Cobalt Aluminum Oxide (NCA) and Nickle Manganese Cobalt Oxide (NMC) cell chemistries at different state of charge (SOC) and temperature combination.

**Figure 6.**
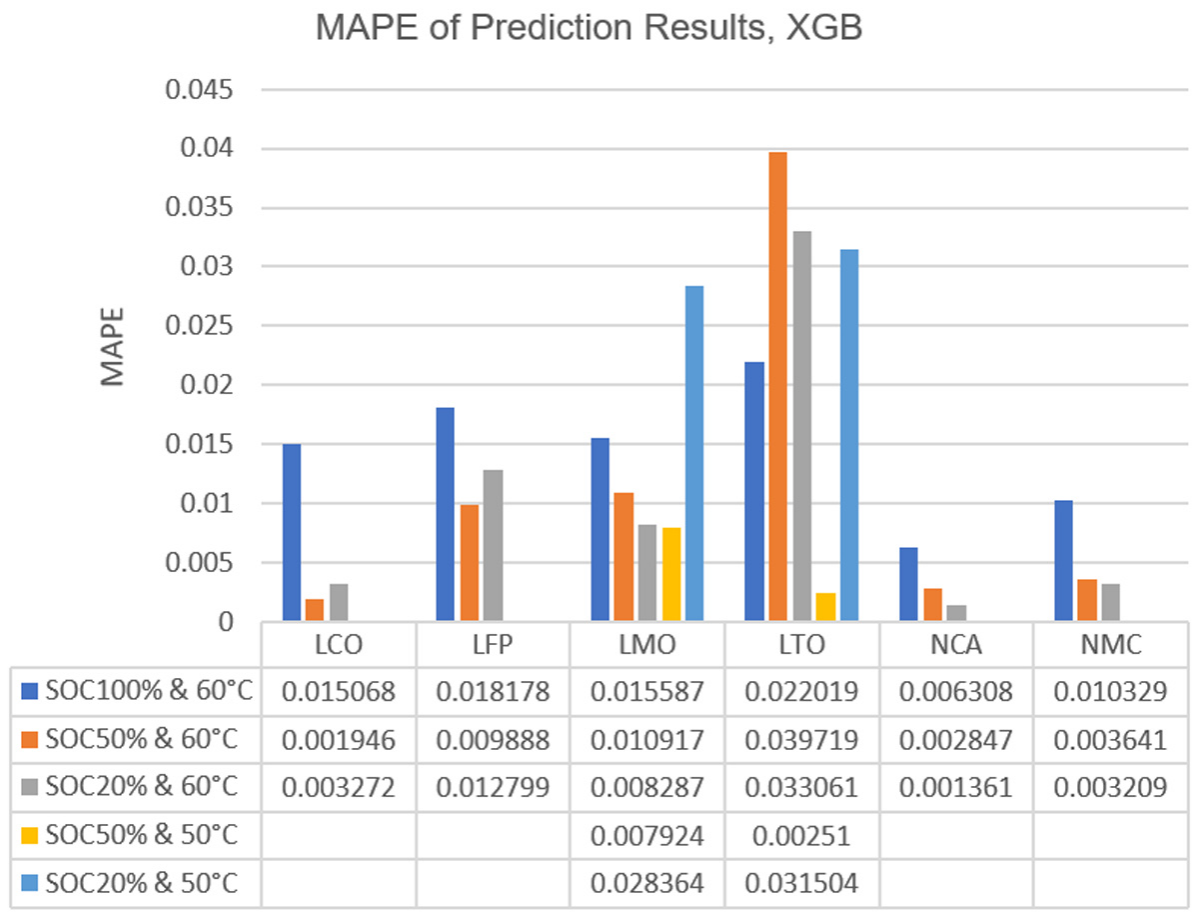
Bar graph of extreme gradient boosting (XGBoost) mean absolute percentage errors (MAPE) of Lithium Cobalt Oxide (LCO), Lithium Iron Phosphate (LFP), Lithium Manganese Oxide (LMO), Lithium Titanium Oxide (LTO), Nickle Cobalt Aluminum Oxide (NCA) and Nickle Manganese Cobalt Oxide (NMC) cell chemistries at different state of charge (SOC) and temperature combination.

In Figure 5, more detailed prediction results of ANN have been presented. The graph demonstrates ANN’s performance at different SOC and temperature combinations. Also, as done with the only-chemistry-focused results, Figure 5 has been supported with the corresponding Ah values to the MAPE values with Table 2. The same kind of visualization and details for XGBoost can be seen in Figure 6 and Table 3.

**Table 2.** Artificial neural network (ANN) mean absolute error values (MAE) of Lithium Cobalt Oxide (LCO), Lithium Iron Phosphate (LFP), Lithium Manganese Oxide (LMO), Lithium Titanium Oxide (LTO), Nickle Cobalt Aluminum Oxide (NCA) and Nickle Manganese Cobalt Oxide (NMC) cell chemistries at different state of charge (SOC) and temperature combinations (in terms of ampere-hour).

SOC% & T	LCO	LFP	LMO	LTO	NCA	NMC
SOC100% & 60°C	0.081378	0.101446	0.145732	0.06983	0.033591	0.106814
SOC50% & 60°C	0.057263	0.079261	0.201832	0.128171	0.083061	0.100742
SOC50% & 50°C	-	-	0.112657	0.115104	-	-
SOC20% & 60°C	0.045575	0.043559	0.072332	0.054119	0.061023	0.063603
SOC20% & 50°C	-	-	0.131381	0.042448	-	-

**Table 3.** Extreme Gradient Boosting (XGB) mean absolute error (MAE) values of Lithium Cobalt Oxide (LCO), Lithium Iron Phosphate (LFP), Lithium Manganese Oxide (LMO), Lithium Titanium Oxide (LTO), Nickle Cobalt Aluminum Oxide (NCA) and Nickle Manganese Cobalt Oxide (NMC) cell chemistries at different state of charge (SOC) and temperature combinations (in terms of ampere-hour).

SOC% & T	LCO	LFP	LMO	LTO	NCA	NMC
SOC100% & 60°C	0.031725	0.025438	0.029895	0.025872	0.014391	0.024037
SOC50% & 60°C	0.004605	0.014662	0.021757	0.042077	0.006978	0.008930
SOC50% & 50°C	-	-	0.015376	0.002688	-	-
SOC20% & 60°C	0.007829	0.019535	0.016149	0.042144	0.003373	0.007844
SOC20% & 50°C	-	-	0.055854	0.04091	-	-

From Figure 5 and Figure 6 it can be seen that, generally, the error increases when algorithms attempt to predict the capacity degradation at 100% SOC or 50°C temperature. The 50°C temperature behaviour may be explained as the majority of the data points are from 60°C conditioning, thus the algorithms expect more drastic capacity drops from their training as occurs in 60°C. However, the reason behind the relatively high errors at 100% SOC is most likely related to the calendar ageing phenomenon’s behaviour itself. The deliberate decision of training the algorithms with all SOC regions available in the data led to the expectation of mild capacity drops under all conditions in them. ANN has its exceptions to this behaviour with its results on LMO, LTO and NCA while XGBoost only shows this exception for LTO.

Another exception to the general behaviour is LTO chemistry results, where the largest error can be seen at 50% SOC and 60°C temperature. In the experiments conducted by THI, this behaviour of the algorithms was explained ^7^. From Figure 4 in 7, it can be seen that LTO is the only chemistry that is subject to a larger capacity degradation at mid-SOC compared to its degradation at the high-SOC level. So ANN’s and XGBoost’s outputs on this are most likely to be more related to chemistry’s calendar ageing behaviour and less dependent on the data sample points or training-test splits.

**Figure 7.**
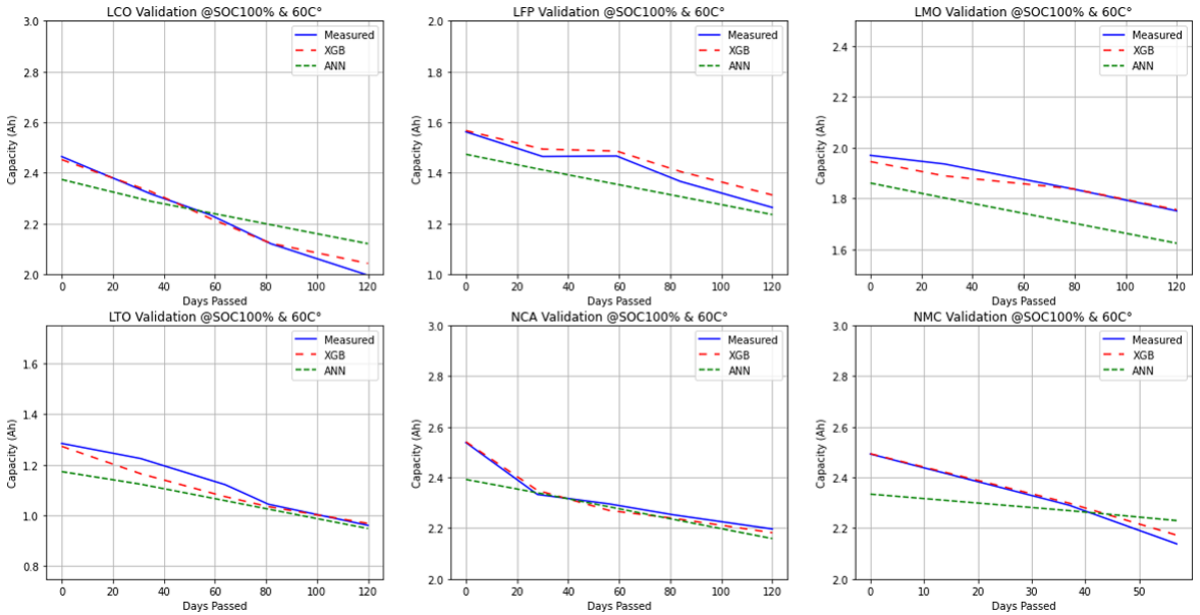
Exemplary continuous time plots of Lithium Cobalt Oxide (LCO), Lithium Iron Phosphate (LFP), Lithium Manganese Oxide (LMO), Lithium Titanium Oxide (LTO), Nickle Cobalt Aluminum Oxide (NCA) and Nickle Manganese Cobalt Oxide (NMC) cell chemistries at 100% state of charge (SOC) and 60°C.

In Figure 7, the continuous time plots of the measured and predicted capacity of cells at 100% SOC and 60°C temperature in terms of passed days plotted and the performances of algorithms in terms of prediction accuracy, can be observed. XGBoost mostly yields outputs that fit the measured data perfectly. Meanwhile, ANN always predicts smaller capacity outputs compared to the cell beginning-of-life (as it should due to the calendar ageing) as the days pass. However, for some chemistries, it may end up with an offset for estimating the degradation, like the results on LMO. Nevertheless, the predicted rate of degradation is the same with the measurement. For LCO and NMC, a pattern of under-estimation for the capacity degradation can be clearly seen. The results fit correctly at the later points for LFP, LTO and NCA chemistries.

While the accuracy of algorithms is an important aspect for an application, the performances in terms of elapsed time for predictions play a huge role as well. Table 4 represents time required for each algorithm to produce the results. From Table 4 it can be seen that ANN can make predictions several times faster than the XGBoost for the whole validation set. However, when an application or the calendar ageing sampling practices are considered, this difference may not have an impact. If the application would be a calendar ageing prediction of an automotive battery pack, executing a function that updates the value of the capacity in 0.002 seconds, 0.0229 seconds (times required to predict the whole validation set on a PC processor) or any other value would not be significant. The reason is probably that this kind of function would have a call time interval in terms of days to write a meaningful capacity change due to the behavior of the phenomenon and when it does get called, how much time is required to update the value and accuracy/computing time trade-off won’t be as significant as is in a SOC estimation application.

**Table 4.** Prediction performances of artificial neural network (ANN) and Extreme Gradient Boosting (XGBoost) algorithms in terms of elapsed time in seconds (whole validation set, PC processor).

Algorithms	Training	Prediction
ANN	0.259736	0.002041
XGB	0.563075	0.022953

The periodic update of the SOC may be required for determining accurate current limits and various other tasks, but periodic updating of the capacity drop due to the calendar ageing won’t be desired during the vehicle operation where embedded software tasks usually run in milli-second intervals. The generated overhead won’t be rewarded with meaningful changes in the capacity. In this work, the elapsed time for predictions is not known for an embedded processor and it is known that the computational efficiency challenges for machine learning applications on embedded systems stand as an obstacle for a wider range adoption of these applications. However, these challenges may be overcome with function calls during the long vehicle park time durations, like nighttime period for an automotive battery pack calendar ageing prediction application, when all application-specific points are considered.

## Conclusion

In this study, the calendar ageing data of six different cell chemistries have been used to train and validate two different machine learning algorithms. One of the expected challenges was XGBoost’s possible overfitting due to the datasets’ length limitation. However, the results demonstrated that the XGBoost algorithm can be used to effectively predict the calendar ageing of most chemistries with significantly minimal mean absolute error. Meanwhile, ANN produces satisfactory results only for LFP, LTO and NCA cell chemistries.

The ability to deploy these models on control systems combined with XGBoost’s satisfying performance on chemistries preferred heavily for the automotive industry (NCA, NMC and LFP), shows that the XGBoost algorithm can be incorporated into the electric vehicle battery application softwares to successfully predict the calendar ageing effects and provide better operation life to electric vehicle batteries. While the same satisfactory use case applies to ANN as well, the prediction performance of XGBoost is far more superior and thus it can establish itself more easily as the first choice. The challenges that stand ahead for these kinds of applications could be the reliance on a large amount of data and computing times ^23^, but machine learning based on big data and cloud computing can be the solution to this particular challenge ^23^ without sacrificing from application specifications mentioned in the previous section.

The models developed in this study are used to predict the capacity degradation resulting from calendar ageing. However, this is only one of the indicators of the effect of calendar ageing as mentioned earlier in this paper. As future work, the other indicator of the calendar ageing in batteries, the resistance increase, can be modeled to be the output of the algorithms. This further work can provide another standpoint to the issue where it can be seen whether or not machine learning algorithms can estimate the calendar ageing via resistance increase with satisfactory performance.

The further work suggestions can be multiplied with some issues mentioned in the results and discussion section, such as the improved and stable correlation between inputs and outputs of the machine learning algorithms for the cobalt-included cell chemistries. This topic should be investigated more since the results on why the usage of cobalt included chemistries is more feasible for calendar ageing prediction via machine learning, will probably be highly interesting and enlightening for many applications. Also, results on the different SOC and temperature combinations discussed based on Figure 5 and Figure 6 can be further investigated. Specifically LTO cells can be researched with a dataset comprising more data points. Current claims are based on educated guesses and quick analyses which revolve around the ideas of data limitations or chemistry specific behaviours based on previous work conducted by 7 and available outputs from the scripts. Further investigations can validate these claims or uncover different root causes.

Lastly, it is worth noting that the results for ANN would be much more promising with denser data, while XGBoost seems to be not affected much by this limitation.

## Data availability

### Underlying data

Zenodo: Lithium-ion battery calendar ageing data - discharge and charge capacity, days passed, temperature and SOC. https://doi.org/10.5281/zenodo.6546753 ^14^

Data are available under the terms of the Creative Commons Attribution 4.0 International license (CC-BY 4.0).
