# Supplementary material for: Calendar ageing modelling using machine learning: an experimental investigation on lithium ion battery chemistries
Source: Open Res Eur. 2023 Feb 22;2:96. Originally published 2022 Aug 12. [Version 2] doi: 10.12688/openreseurope.14745.2 (PMC10446031; doi:10.12688/openreseurope.14745.2)
Supplement: Peer review history

# Peer review: Reviewer response for version 2

**DOI:** 10.21956/openreseurope.16982.r30873

**Published:** 2023-03-02

**Authors:** Min Ye

**Affiliations:** 1 National Engineering Laboratory for Highway Maintenance Equipment, School of Construction Machinery, Chang’an University, Xi’an, China

The revision was perfect.

Is the study design appropriate and does the work have academic merit?

Yes

Is the work clearly and accurately presented and does it cite the current literature?

Yes

If applicable, is the statistical analysis and its interpretation appropriate?

Yes

Are all the source data underlying the results available to ensure full reproducibility?

Partly

Are the conclusions drawn adequately supported by the results?

Yes

Are sufficient details of methods and analysis provided to allow replication by others?

Partly

Reviewer Expertise:

energy management system

I confirm that I have read this submission and believe that I have an appropriate level of expertise to confirm that it is of an acceptable scientific standard.

# Peer review: Reviewer response for version 1

**DOI:** 10.21956/openreseurope.15927.r30383

**Published:** 2022-12-02

**Authors:** Min Ye

**Affiliations:** 1 National Engineering Laboratory for Highway Maintenance Equipment, School of Construction Machinery, Chang’an University, Xi’an, China

The authors used the machine learning algorithms, Extreme Gradient Boosting (XGBoost) and artificial neural network (ANN) to predict calendar aging data for six lithium-ion batteries. The prediction results show that XGBoost error rate and fitting performance are more suitable for EV battery calendar aging prediction applications. Hence, the paper may be value of being accepted after carefully revisions.

 Some specific comments are as follows:

1. In terms of literature, this year's references on XGBoost and battery aging models should be appropriately cited.
2. Please make sure that all the variables are in italics and should be defined in the annotations accordingly.
3. The diagram of the full paper should be clear, with detailed abscissa ordinate notes and units. For example, the abscissa in Figure 7 should be added to the units.
4. In the introduction, the organization and the main contributions of the paper are missing, please add relevant content accordingly.
5. Figure 1 is too simple. Some information about the paper topic should be added. Or else delete it.
6. In figure 2, % should be added into the Soc value, for example soc < 75%.
7. It is better to express the number in percentage, such as MAE and MAPE and other results instead of decimal value. The format will be more clear.

Is the study design appropriate and does the work have academic merit?

Yes

Is the work clearly and accurately presented and does it cite the current literature?

Yes

If applicable, is the statistical analysis and its interpretation appropriate?

Yes

Are all the source data underlying the results available to ensure full reproducibility?

Partly

Are the conclusions drawn adequately supported by the results?

Yes

Are sufficient details of methods and analysis provided to allow replication by others?

Partly

Reviewer Expertise:

energy management system

I confirm that I have read this submission and believe that I have an appropriate level of expertise to confirm that it is of an acceptable scientific standard, however I have significant reservations, as outlined above.

# Response

**Published:** 2022-12-12

**Authors:** Furkan Metin Turgut

**Affiliations:** AVL Research and Engineering Turkey, Turkey

Hello Prof. Ye, I have some questions about your comments. I would appreciate if we could eloborate some of them.

 - You mentioned annotations, do you think there should be a nomenclature section where we explain the variables ?

 - You wanted an organization and main contributions part in the paper. However, initially even i added that ORE colleagues wanted me to scratch it. Instead, those information has been added to the ORE webpage of the paper

 - Prof. Ye, we spent a lot of time on data availability under the guidance of ORE colleagues. As a result, we added really explanatory and rich data to Zenodo. It is the data we use to produce the results. Can you explain why you answered data availability question as "partly" and what can we do for you to change this ?

 - Regarding the other "partly" answer, Prof. Ye, we are a R&D company but usually providing services as an automotive industry partner. We cannot exactly share and give away the codes and other tools we used to produce the results because they have been stemmed from customer projects of AVL. Also, investments have been made to companies and employees to accumulate the know-how. You answered the question about the detail of the methodology as "partly" but what can we do for you to reconsider this? Can you eloborate? Other than these, I am already finished with your other remarks,

 - You mentioned detailed abscissa for Figure 7, I added the data sample points to the plot (I am assuming this is what you mean by abscissa and is there any other figure needing this other than Figure 7. I only did it for Figure 7) - I deleted the Figure 1 - Converted each MAPE result to their % equivalent - Added % to the SOC values in Fig. 2.

# Peer review: Reviewer response for version 1

**DOI:** 10.21956/openreseurope.15927.r29904

**Published:** 2022-08-23

**Authors:** Suleyman Tosun

**Affiliations:** 1 Department of Computer Engineering, Hacettepe University, Ankara, Turkey

The paper presents a calendar aging modeling method that uses two different machine learning algorithms; namely,  Extreme Gradient Boosting (XGBoost) and artificial neural network (ANN).

 In the introduction, the authors explain why the calendar aging modeling is essential for batteries of different types. Therefore, they show how this work fulfills the necessity of modeling calendar aging. 

 As a methodology, they use XGBoost and ANN machine learning algorithms. XGBoost is a decision tree-based machine learning algorithm. The authors give the decision tree of the algorithm using 10 different parameters. The algorithm also gives decisions even if some of the data are missing when tracking the branches of the tree. The second method is ANN, which is the most commonly used machine learning algorithm. The selection of these algorithms is suitable for the problem since both algorithms have been proven to have good prediction capabilities. 

 The authors compare these two methods in terms of mean absolute error (MAE) and percentile variation mean absolute percentage error (MAPE). The analysis of the results shows that the XGBoost has a better prediction than ANN for all types of batteries in terms of the determined MAE and MAPE results. The results seem accurate and the method can be applied to commercial tools.

 Overall, the paper gives the necessary information for a reader who would like to implement and experience the algorithms presented in the article. 

 My only suggestion is that the paper needs some grammatical and punctuation corrections. For example, I recommend not to use informal "don't" or "doesn't". Instead, the words "do not" or does not" should be used. Additionally, there are some sentences that need to be checked again for correct punctuation. You can use commercial grammar tools for that.

Is the study design appropriate and does the work have academic merit?

Yes

Is the work clearly and accurately presented and does it cite the current literature?

Yes

If applicable, is the statistical analysis and its interpretation appropriate?

Yes

Are all the source data underlying the results available to ensure full reproducibility?

Yes

Are the conclusions drawn adequately supported by the results?

Yes

Are sufficient details of methods and analysis provided to allow replication by others?

Yes

Reviewer Expertise:

Computer engineering and computer science

I confirm that I have read this submission and believe that I have an appropriate level of expertise to confirm that it is of an acceptable scientific standard.
